# A cuckoo in wolves' clothing? Chemical mimicry in a specialized cuckoo wasp of the European beewolf (Hymenoptera, Chrysididae and Crabronidae)

**DOI:** 10.1186/1742-9994-5-2

**Published:** 2008-01-11

**Authors:** Erhard Strohm, Johannes Kroiss, Gudrun Herzner, Claudia Laurien-Kehnen, Wilhelm Boland, Peter Schreier, Thomas Schmitt

**Affiliations:** 1 University of Regensburg, Institute of Zoology, D-93040 Regensburg, Germany; 2 University of Würzburg, Department of Animal Ecology and Tropical Biology, Am Hubland, 97074 Würzburg, Germany; 3 University of Bonn, Zoological Institute, Poppelsdorfer Schloss, 53115 Bonn, Germany; 4 University of Bielefeld, Behavioural Biology, 33501 Bielefeld, Germany; 5 Max Planck Institute for Chemical Ecology, Department for Bioorganic Chemistry, 07745 Jena, Germany; 6 University of Würzburg, Department of Food Chemistry, Am Hubland, 97074 Würzburg, Germany; 7 University of Freiburg, Institute of Biology I (Zoology), Hauptstr. 1, 79104 Freiburg i. Br., Germany

## Abstract

**Background:**

Host-parasite interactions are among the most important biotic relationships. Host species should evolve mechanisms to detect their enemies and employ appropriate counterstrategies. Parasites, in turn, should evolve mechanisms to evade detection and thus maximize their success. Females of the European beewolf (*Philanthus triangulum*, Hymenoptera, Crabronidae) hunt exclusively honeybee workers as food for their progeny. The brood cells containing the paralyzed bees are severely threatened by a highly specialized cuckoo wasp (*Hedychrum rutilans*, Hymenoptera, Chrysididae). Female cuckoo wasps enter beewolf nests to oviposit on paralyzed bees that are temporarily couched in the nest burrow. The cuckoo wasp larva kills the beewolf larva and feeds on it and the bees. Here, we investigated whether *H. rutilans *evades detection by its host. Since chemical senses are most important in the dark nest, we hypothesized that the cuckoo wasp might employ chemical camouflage.

**Results:**

Field observations suggest that cuckoo wasps are attacked by beewolves in front of their nest, most probably after being recognized visually. In contrast, beewolves seem not to detect signs of the presence of these parasitoids neither when these had visited the nest nor when directly encountered in the dark nest burrow.

In a recognition bioassay in observation cages, beewolf females responded significantly less frequently to filter paper discs treated with a cuticular extract from *H. rutilans *females, than to filter paper discs treated with an extract from another cuckoo wasp species (*Chrysis viridula*). The behavior to paper discs treated with a cuticular extract from *H. rutilans *females did not differ significantly from the behavior towards filter paper discs treated with the solvent only.

We hypothesized that cuckoo wasps either mimic the chemistry of their beewolf host or their host's prey. We tested this hypothesis using GC-MS analyses of the cuticles of male and female beewolves, cuckoo wasps, and honeybee workers. Cuticle extracts of *Hedychrum nobile *(Hymenoptera: Chrysididae) and *Cerceris arenaria *(Hymenoptera: Crabronidae) were used as outgroups. There was little congruence with regard to cuticular compounds between *H. rutilans *females and honeybees as well as females of *C. arenaria *and *H. nobile*. However, there was a considerable similarity between beewolf females and *H. rutilans *females. Beewolf females show a striking dimorphism regarding their cuticular hydrocarbons with one morph having (*Z*)-9-C25:1 and the other morph having (*Z*)-9-C27:1 as the major component. *H. rutilans* females were more similar to the morph having (Z)-9-C27:1 as the main component.

**Conclusion:**

We conclude that *H. rutilans *females closely mimic the composition of cuticular compounds of their host species *P. triangulum*. The occurrence of isomeric forms of certain compounds on the cuticles of the cuckoo wasps but their absence on beewolf females suggests that cuckoo wasps synthesize the cuticular compounds rather than sequester them from their host. Thus, the behavioral data and the chemical analysis provide evidence that a specialized cuckoo wasp exhibits chemical mimicry of the odor of its host. This probably allows the cuckoo wasp to enter the nest with a reduced risk of being detected by olfaction and without leaving traitorous chemical traces.

## Background

The interaction between hosts and parasites or parasitoids is one of the most important forces driving evolutionary and ecological processes [1]. In order to reduce the impact of parasitoids, host species may evolve mechanisms to detect their enemies and employ adequate counterstrategies [2-5]. Parasitoids, in turn, are selected to evolve mechanisms that reduce the probability of being detected by their hosts to circumvent such countermeasures. This sets the stage for repeated cycles of adaptations and counteradaptations ("evolutionary arms race" [6-10]) between hosts and parasites, especially if the parasitoid is highly specialized on a single host species and has a large impact on host fitness [2,11,12].

Progeny of brood caring bees and wasps are particularly susceptible to parasitism [13-15]. Females of these species store large amounts of valuable nutrients as larval provisions in brood cells. These valuable resources attract a variety of brood parasites, either cleptoparasites that reduce the amount of resources available to the host's progeny or parasitoids that obligatorily kill the host larvae. Mostly, females of these parasitic species have to enter the nest or the brood cell to deposit eggs or larvae. Thus, the traces that are left by female brood parasites might be detected by the host. As a result, the hosts might abandon the nests or remove or destroy eggs of brood parasites [5,16]. Brood parasites might also be encountered in the nest by the host and might be driven away, injured, or even killed (E. Strohm, unpubl. observations). Since insects heavily rely on their chemical senses for any kind of recognition or localization process [1,17,18], concealment of a brood parasite's actual or previous presence will require chemical camouflage (compounds sequestered from the host or the host's nest) or chemical mimicry (compounds synthesized by the mimic, definitions *sensu *[19]). In this study, we investigated the interaction between a hunting wasp, the European beewolf, *Philanthus triangulum *(Hymenoptera, Crabronidae) and its highly specialized brood parasitoid *Hedychrum rutilans *(Hymenoptera, Chrysididae). We investigated the following questions: Are cuckoo wasps (*H. rutilans*) detected and recognized by beewolf females at all? Is there a difference in host response towards the cuckoos outside and inside the nest and do the cuticular hydrocarbons play a role for the detection of the cuckoo wasps inside the nest? Is the chemical composition of the cuticular hydrocarbons of *H. rutilans *females similar to their host or to their host's prey?

Females of the European beewolf hunt honeybee workers (Hymenoptera, Apidae) as food for their progeny. Several paralyzed bees are temporarily couched in the main burrow (up to 1 m long) of the underground nest (see [20] for details on nest architecture). Eventually, the female closes the nest entrance, excavates a side burrow and a terminal brood cell, brings in one to six paralyzed bees, and oviposits on one of the bees [21]. Thereupon, she carefully closes the side burrow and subsequently has no contact to her progeny.

The cuckoo wasp, *H. rutilans*, is a specialized brood parasitoid of the genus *Philanthus *[16,22]. However, since in Central Europe only one member of the genus, *P. triangulum*, is fairly abundant, *H. rutilans *is effectively monospecific in this region. This considerable degree of specialization is expressed by the unique oviposition strategy of *H. rutilans*. Most chrysidid wasps oviposit into the brood cell of their hosts at a defined stage of the provisioning cycle or after the brood cell has been finally closed [22]. In beewolves, however, the brood cell is excavated only after the female has brought in the bees and the nest entrance has been closed. Thus, the nest is blocked up and the female is attendant until the brood cell is finally closed. This leaves little opportunity for a cuckoo wasp to deposit an egg in the brood cell. As a consequence, *H. rutilans *females pursue two alternative strategies. Either they rapidly pounce and oviposit on a paralyzed bee when the female alights with its prey and enters the burrow ([23], E. Strohm unpubl. observation), or *H. rutilans *females wait in front of the nest until the host female leaves to forage and then enter the burrow and oviposit on the paralyzed bees that are temporarily couched there [22]. Thus, *H. rutilans *use the paralyzed bees as a Trojan horse to bring the egg into the brood cell. The latter seems to be the much more frequent mechanism. The mobile larva of *H. rutilans *climbs onto the beewolf larva, kills it, and feeds on the host larva and the bees. Thus, infestation by *H. rutilans *inevitably leads to a fitness reduction of the host. *H. rutilans *is considered to be the most important brood parasite of *P. triangulum*. The rate of parasitism varies between 3% and more than 30% ([4,24-26], E. Strohm, unpubl. data). *H. rutilans *might even drive local aggregations of *P. triangulum *to extinction [25].

In both oviposition strategies, detection of the cuckoo wasp female by the beewolf female might decrease the cuckoo wasp's success. First, when encountered in the nest, cuckoo wasps might be carried to the nest entrance by beewolf females and thrown out [27]. Mostly, cuckoo wasps are not severely harmed due to the solidity and strong sculpturing of their cuticle and their ability to adopt a rolled-up defensive posture that protects the most vulnerable parts of the body (legs, mouthparts, antennae [22,28]). Nevertheless, the wings are rather unprotected and might be injured by a beewolf female. Second, if beewolves females detect signs of the presence of cuckoo wasps they might remove bees from the nest that have possibly been parasitized [25]. Thus, a cuckoo wasp should avoid detection to minimize wastage of time and investment. This means that cuckoo wasp females should avoid detection when they are encountered by a host female in the nest. However, it would probably be even more important for the cuckoo wasps not to leave any detectable traces of their presence when they had entered the nest and oviposited on a paralyzed honeybee.

In order to assess whether beewolf females respond to the presence of *H. rutilans *females at all we observed the behavioral interactions outside of the nest. To test whether *H. rutilans *females employ chemical camouflage inside the nest we conducted to sets of behavioral experiments. First, in observation cages we recorded the interaction of the cuckoo wasps with beewolf females inside the nest burrow. Second, we conducted a recognition bioassay by assessing the response of beewolf females towards filter discs treated with different extracts: solvent only, cuticular extracts of another chrysidid, *Chrysis viridula*, and cuticular extracts of *H. rutilans*. We predicted that beewolf females should ignore the discs treated with solvent only (negative control), they should respond to the discs treated with *C. viridula *extract (positive control) and they should not (or only weakly) respond to *H. rutilans *extracts.

There are two evolutionary options for *H. rutilans *femalesto avoid olfactory detection by beewolves. First, cuckoo wasps could mimic the odor of the honeybees that are temporarily couched in the main burrow. Second, *H. rutilans *females might mimic their beewolf host. We consider the imitation of the cuticular compounds of the beewolf host the better alternative, since the host's hydrocarbon profile can be found all over the nest walls due to the digging activity (Kroiss and Strohm unpubl. data) and also on the honeybees. This is because in order to prevent the paralyzed bees from molding they are treated by the beewolf females with a secretion from the postpharyngeal gland that is identical to the beewolves' cuticular hydrocarbons [29-31].

To assess both alternatives, we analyzed the composition of cuticular compounds of beewolf females, cuckoo wasps, and honeybees. Furthermore, we included beewolf males as the *a priori *most similar group to beewolf females and, thus, a crucial comparison for assessment of this hypothesis. To control for the possibility that chrysidids and crabronids have similar patterns of cuticular hydrocarbons by chance we also analyzed closely related species: the chrysidid wasp *Hedychrum nobile *and its crabronid host *Cerceris arenaria *(subfamily Philanthinae, a weevil hunting wasp). A reasonable null hypothesis for the resemblance among the species under study might be based on their phylogenetic relationship. Crabronids and apids are closely related and constitute the superfamily Apoidea, whereas chrysidids branch off very early [32,33]. Thus, the null hypothesis would predict that the cuticular profiles of beewolf females should be most similar to conspecifics males and *Cerceris *females, fairly similar to honeybees, and least similar to cuckoo wasps. Accordingly from a phylogenetic point of view, the chemical profiles of the two congeneric chrysidid species should be most similar to each other.

## Results

### Are cuckoo wasps detected by beewolf females outside the nest?

Cuckoo wasps flew over the beewolf nesting site and selectively landed on the mounds of beewolf nests. During the 54 hours of observation we recorded 1024 landings of *H. rutilans *on beewolf nest mounds. In 259 cases (25.3%), cuckoo wasps flew off after ≤ 4 seconds. In 765 cases (74.7%) they remained on or in the vicinity of the nest mound for ≥ 4 seconds, the duration of these stays was 74 ± 250 s (median = 11 s). During these stays the cuckoo wasps moved on the nest mound, vigorously antennating the surface. During prolonged stays at the nest, cuckoo wasps often moved to shaded areas and sometimes even placed themselves under some nearby structures like leaves. In 37 of the 765 cases the entrance was open and cuckoo wasps entered the nest for 13 – 270 s (mean: 118 ± 133 s, median: 60 s). In the remaining 728 cases the entrance was closed. Nevertheless, in 41 of these cases cuckoo wasps tried to dig through the closure; in 29 cases they abandoned digging after some time. In the 12 remaining cases they dug through the nest closure and stayed in the nest for 14 to 1263 s (mean: 384 ± 421 s, median 213 s). During the observation time we observed 89 beewolf females returning with a paralyzed honeybee and entering their nests. In four of these cases we observed attempts of cuckoo wasp females to attach to a honeybee that was carried by a beewolf female while entering the nest with its prey. In all four cases, the female detected the parasitoid and drove it away. In another 11 cases (of the 89) cuckoo wasps were driven away by homing beewolf females although they did not approach the prey laden female to oviposit. In one of these cases the beewolf female grasped the cuckoo wasp with her mandibles. Sometimes (46 cases of 765 cases), cuckoo wasps were driven away from a nest mound by the approach of another cuckoo wasps. Although in one of the 89 cases the cuckoo wasp was in the nest when the beewolf female returned with a bee, it was not thrown out of the nest.

### Are cuckoo wasps detected inside nests?

#### a) Experiment I

In observation cages in the laboratory, *H. rutilans *females (N = 7) were observed to enter beewolf nests (N = 6 beewolf nests) and oviposit on the couched bees (N = 4). Although in five cases the beewolf female entered the nest while a cuckoo wasp was present and came close to (less than 2 cm, N = 5) or even passed (N = 3) the cuckoo wasp in the burrow, the host female did not show any signs of detection of the brood parasitoid or disturbance. Notably, the cuckoo wasp either ran to a distant part of the nest when a beewolf female approached or it remained motionless at the periphery of the nest burrow until the female had passed.

#### b) Experiment II

**Figure 1:**
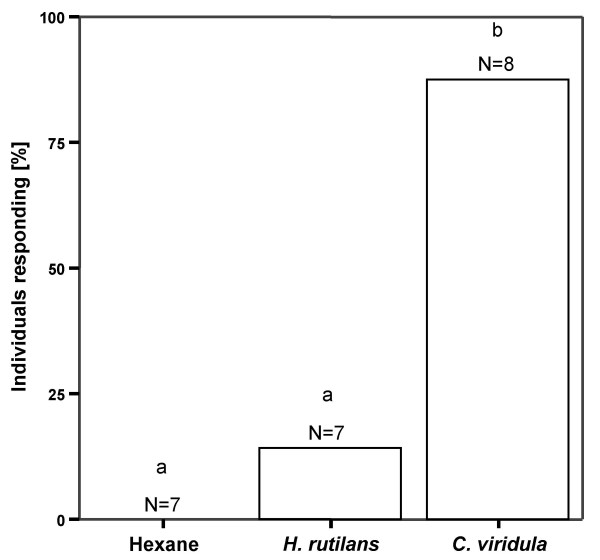
**Recognition bioassay**. Percentage of beewolf females showing a response towards filter paper discs treated with hexane (negative control, left), cuticular extract of *H. rutilans *females (middle), and cuticular extract of *C. viridula *females (right). Different letters above the bars indicate significant differences between groups (Fisher's exact test: P < 0.05).

In the recognition bioassay (Figure 1), beewolf females always responded to the positive control (*C. viridula *extract), they never responded to the negative control (solvent only), and they only rarely responded to the test discs (*H. rutilans *extract). The difference in response frequency between *H. rutilans *and *C. viridula *extracts was significant (Fisher's exact test: P = 0.0101, Figure 1) whereas there was no statistical difference between *H. rutilans *extracts and the negative control (Fisher's exact test: P = 1, Figure 1). This result shows that the chemical signal consisting of cuticular extracts of *H. rutilans *elicits much weaker behavioral responses in beewolf females than extracts of a closely related chrysidid species. Thus, we hypothesized that *H. rutilans *females are chemically camouflaged.

### Are cuckoo wasps chemically cloaked?

The GC-MS analyses revealed alkanes, alkenes, and mono- and dimethylalkanes as the predominant hydrocarbons in all species. We found between 12 and 34 substances on the cuticles of the five species (Table 1). *H. rutilans *females shared 10 compounds with beewolf females, 9 with beewolf males, 13 with *H. nobile*, 11 with honeybees, and 14 with *C. arenaria *females.

**Table 1:** Relative peak area of cuticular compounds.

	*A. mellifera *workers	*P. triangulum *males	*P. triangulum *females	*H. rutilans *females	*H. nobile *females	*C. arenaria *females
C21	0.399	-	-	-	0.194	-
C22	0.128	-	-	-	-	-
(Z)-11-Eicosen-1-ol	0.471	-	-	-	-	-
(Z)-9-C23:1	2.81	0.261	-	-	0.884	-
(Z)-7-C23:1	0.301	-	-	-	0.210	-
C23	22.4	16.5	10.2	10.1	3.56	0.138
11-,9-MeC23	-	-	-	-	2.70	-
7-MeC23	-	-	-	-	2.69	-
5-MeC23	-	-	-	-	0.289	-
3-MeC23	-	-	0.387	1.10	1.01	-
C24:1	1.15	0.261	-	-	-	-
C24	0.508	0.756	-	-	0.213	0.188
(Z)-9-C25:1	5.31	34.5	40.8	11.0	9.55	0.038
(Z)-7-C25:1	0.233	-	-	10.6	3.10	0.087
C25	24.8	9.49	5.80	20.3	21.0	32.7
13-,11-,9-MeC25	0.043	-	-	1.95	9.41	1.22
7-MeC25	0.010	-	-	0.975	8.33	0.188
5-MeC25	-	-	-	0.489	1.40	0.576
7,11-diMeC25, 3-MeC25	-	-	-	-	0.710	0.254
C26:1	-	0.281	0.443	0.211	-	-
C26	0.384	-	-	-	0.764	0.531
13-,12-,11-,10-,9-,8-,7-MeC26	-	-	-	-	0.639	0.370
16-Pentacosen-8-one	-	-	0.307	-	-	-
(Z)-9-C27:1	1.93	2.16	31.2	13.9	0.683	0.434
(Z)-7-C27:1	-	-	-	6.32	0.588	5.42
C27	12.9	5.15	2.51	10.6	10.9	7.12
13-,11-,9-,7-MeC27	0.368	-	-	1.58	15.0	25.7
5-MeC27	-	-	-	-	0.311	0.073
9,13-diMeC27	-	-	-	-	0.412	0.320
7,11-diMeC27, 3-MeC27	-	-	-	-	-	0.223
5,9-,5,11-diMeC27	-	-	-	-	-	0.643
C28	0.154	-	-	-	0.249	0.197
14-,13-,12-,11-,10-,9-,8-,7-MeC28	-	-	-	-	0.286	0.546
18-Heptacosen-10-one	-	-	0.527	-	-	-
(Z)-9-C29:1	0.341	0.047	1.38	0.571	-	0.771
7-C29:1	0.653	-	-	-	-	5.00
C29	4.53	3.49	4.01	8.36	1.79	4.00
13-,11-,9-,7-MeC29	0.250	-	-	-	2.42	11.9
5-MeC29	-	-	-	-	-	0.247
7,11-diMeC29+3-MeC29	-	-	-	-	-	0.031
5,13-,5,11-,5,9-diMeC29	-	-	-	-	-	0.083
C30	-	-	-	-	-	0.055
14-,13-,12-,11-,10-,9-,8-MeC30	-	-	-	-	-	0.080
C31:1	4.78	0.553	-	-	-	0.046
C31:1	0.635	0.491	-	-	-	0.098
C31	5.41	1.85	2.47	1.98	--	0.246
15-,13-,11-,9-,7-MeC31	-	-	-	-	0.635	0.490
C33:1	0.762	-	-	-	-	-
C33:1	7.87	11.7	-	-	-	-
C33	0.377	12.6	-	-	-	-

Relative peak area (in percent, not transformed) of compounds on the cuticle of *A. mellifera *workers (N = 8), *P. triangulum *males (N = 8), *P. triangulum *females (N = 7), *H. rutilans *females N = 13), *H. nobile *females (N = 4), and *C. arenaria *females (N = 4).

The cuticles of honeybees contained a varying proportion of saturated and unsaturated hydrocarbons with a chain lengths ranging from C25 to C33. The profile of beewolf females is characterized by a very high proportion of unsaturated hydrocarbons with individuals showing either (*Z*)-9-C25:1 or (*Z*)-9-C27:1 as the main peak (for the source and possible function of this chemical dimorphism see [29-31,34-36]). Cuckoo wasps also show relatively large amounts of the unsaturated C25:1 and C27:1. In contrast to beewolves where individuals had only large proportions of one of these unsaturated compounds, *H. rutilans *females showed fairly large proportions of both. Furthermore, cuckoo wasps had similar proportions of the (*Z*)-7- and the (*Z*)-9-isomers of both substances (Table 1).

**Figure 2:**
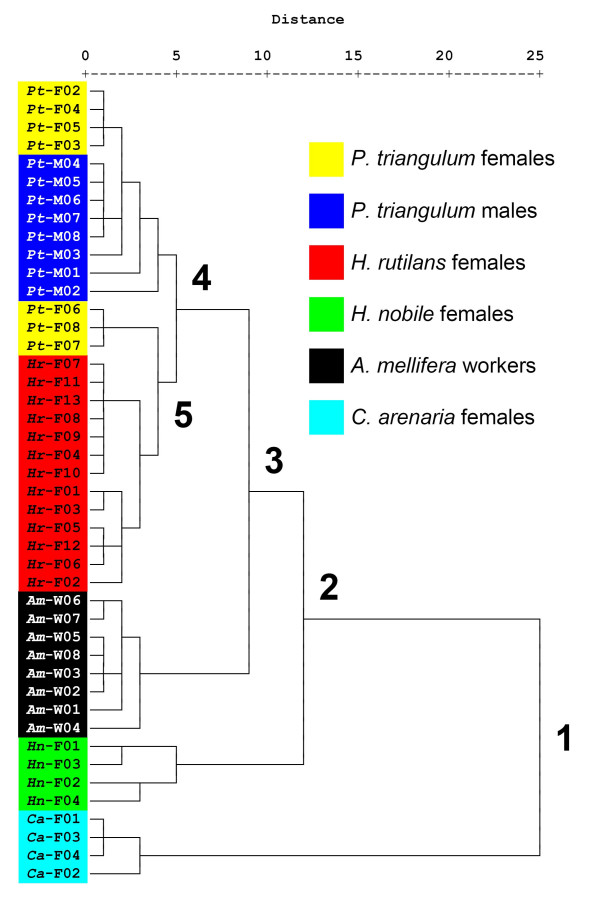
**Dendrogram based on the cluster analysis of the cuticular compounds**. Included are individual *P. triangulum *females (= *Pt*-F), *P. triangulum *males (= *Pt*-M), *H. rutilans *females (= *Hr*-F), *A. mellifera *workers (= *Am*-W), *C. arenaria *females (= *Ca*-F), and *H. nobile *females (= *Hn*-F). Numbers after the species label indicate the different individuals. Numbers in the dendrogram indicate the first 5 bifurcations (see text).

A cluster analysis based on the proportions of cuticular hydrocarbons as revealed by GC-MS (Figure 2) clearly separated *C. arenaria *females from all other species in the first bifurcation. The second bifurcation separated *H. nobile *from the honeybee workers, the beewolves, and *H. rutilans*. The remaining individuals were subdivided by bifurcation three into the honeybees on the one side and the beewolves and *H. rutilans *on the other side. Among beewolves and *H. rutilans*, however, the distinction was less clear-cut. The fourth bifurcation separated a group consisting of all male beewolves and four beewolf females (that shared (*Z*)-9-C25:1 as the major component) from a group consisting of the other three beewolf females (that shared (*Z*)-9-C27:1 as the major component) and *H. rutilans *females. Thus, the cuckoo wasps most closely resemble beewolf females exhibiting (*Z*)-9-C27:1 as their major cuticular compound. Only bifurcation five separated the *H. rutilans *females from the three beewolf females of that cluster. According to this analysis, *H. rutilans *females are considerably more similar to beewolf females than to females of the closely related *H. nobile*, and are about as similar to beewolf females as beewolf males.

**Figure 3:**
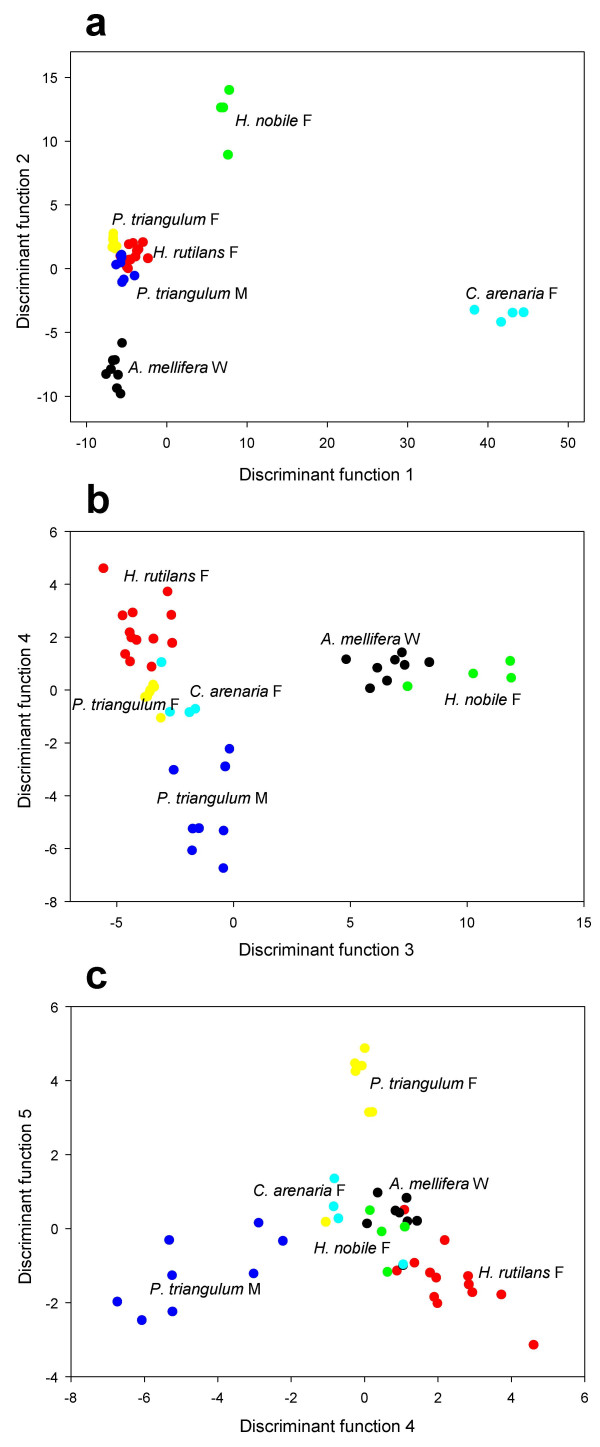
**Discriminant analysis of the cuticular compounds**. Included are individual *P. triangulum *females (yellow), *P. triangulum *males (blue), *H. rutilans *females (red), *A. mellifera *workers (black), *C. arenaria *females (turquoise), and *H. nobile *females (green): Representation of the six groups for five discriminant functions a: Discriminant functions (DF) 1 and 2; b: DF 3 and 4; c: DF 4 and 5. The analysis is based on 8 factors revealed by the principal component analysis.

The discriminant analysis, that followed the principal component analysis, calculated five discriminant functions that resulted in a complete separation of the six groups (Wilk's Λ < 0.001, d.f. = 40, P < 0.001; Figure 3, Table 2). Discriminant function 1 represented 75.8% of the variance and clearly separated females of both *C. arenaria *and *H. nobile *from the other groups. Discriminant function 2 represented 10.8% of the variance and separated honeybees from a group consisting of male and female beewolves and *H. rutilans *females. Discriminant functions 3 (10.1% of the variance) and 4 (2.2% of the variance) separated beewolf males from beewolf females. Only discriminant function 4 and discriminant function 5 (1.1% of the variance) separated beewolf females from *H. rutilans *females. Thus, although *H. rutilans *females can be separated from beewolf females using GC-MS and discriminant analysis, the amount of variance that allows this separation is very small.

**Table 2:** Group centroids for the discriminant analysis.

	discriminant function				
species	1 (75.8%)	2 (10.8%)	3 (10.1%)	4 (2.2%)	5 (1.1%)

*H. rutilans *females	-4.239	1.064	-3.980	2.312	-1.359
*P. triangulum *females	-6.591	2.100	-3.512	-0.187	3.498
*P. triangulum *males	-5.507	0.170	-1.122	-4.593	-1.205
*A. mellifera *workers	-6.414	-7.978	6.651	0.874	0.283
*C. arenaria *females	41.874	-3.566	-2.335	-0.332	0.316
*H. nobile *females	7.279	12.050	10.360	0.583	-0.175

Given are the respective group means of the five discriminant functions (percent explained variance).

## Discussion

Beewolf females often attacked and evicted *H. rutilans *when they encountered them in front of their nest. This seems to be the rule for interactions between hosts and chrysidids although Linsenmaier [37] reported that there are also cases where chrysidids do not elicit antagonistic behavior by their hosts. Prolonged stays at hosts' nests as observed in *H. rutilans*, have also been reported for other chrysidids [22,37,38]. Staying in vicinity of the host nest might allow the chrysidids to adjust the timing of oviposition to the most suitable stage of the provisioning cycle or to enter the nest in the absence of the host female. That *H. rutilans *females placed themselves under some cover (e. g. leaves) during prolonged stays might, besides the reduction of water loss, represent an attempt to hide themselves from the host females. Other chrysidid species also seem to hide near the entrance of a host nest and inspect the nest or brood cell after the host female has deposited provisions and departed for a new foraging flight [37]. This suggests that, similar to beewolves, most host species might recognize cuckoo wasps visually outside the nest. Since most chrysidids are brightly colored (see e.g. drawings in [37]) this is not surprising.

However, there was no evidence that *H. rutilans *females were recognized by beewolf females in their nests although the nest owners approached the chrysidids several times. In contrast to Olberg's few reported cases [27], we could never observe that chrysidids were thrown out of the nest by beewolf females neither in the study population in the field, nor in observation cages in the laboratory, nor during prolonged observations of beewolf nest aggregations as part of another study [4]. Possibly, different populations show varying local adaptations in the defense of the parasitoid or this behavior is extremely rare. However, we can not exclude that the chrysidids that Olberg [27] saw were not *H. rutilans*. Anyhow, our observations suggest that the *H. rutilans *females are often not detected in the nests. This finding is supported by the recognition bioassay. Filter paper discs treated with an extract of *H. rutilans *were recognized significantly less frequently than paper discs treated with an extract of the "alien" cuckoo wasp *C. viridula*. This suggests that the properties of the cuticular hydrocarbons of *H. rutilans *allow the cuckoo wasp to avoid recognition either when encountered inside the nest or after visiting the nest for inspection or oviposition. Our observations furthermore suggest that in the vast majority of cases *H. rutilans *females enter the nest when the female is out foraging and that they are only rarely directly encountered by the nest owner. Thus, it seems most important not to leave traces in the burrow during inspection of the nest or oviposition.

There are numerous reports of parasites of social species that gain access to their hosts' nests and protection from attacks mainly by chemical camouflage and more rarely chemical mimicry [19,39-45] see also [46,47] for examples of chemical mimicry of sex pheromones in sexually deceptive orchids). Hydrocarbons are considered to represent the principal cues for nestmate recognition in social bees and wasps [41] and are most probably also involved in nest identification and species recognition in solitary species [48]. In our analysis, the GC-MS profiles of the cuticular hydrocarbons of *H. rutilans *and beewolf females show considerable similarity. At least, the null hypothesis based on the phylogenetic relationship of a closer resemblance between the two *Hedychrum *species as well as the two philanthine species was clearly contradicted. Our preliminary analysis of other species of chrysidids apart from *H. nobile *showed also distinct differences to the profile of *H. rutilans *(J. Kroiss, T. Schmitt, P. Schreier, E. Strohm, unpubl. data). Furthermore, other aculeate Hymenoptera show considerably different compositions of cuticular compounds [49-52]. This clearly contradicts a general similarity among all Hymenoptera or all aculeates or between chrysidids and crabronids. The profiles of the *H. rutilans *females were as close as or even closer to beewolf females than the profiles of beewolf males. Together with the behavioral tests, this provides strong evidence that *H. rutilans *females are chemically cloaked. This will help to reduce the probability of detection during or -more importantly- after their presence in the nest.

*A priori*, chemical camouflage and mimicry seem unlikely to evolve in a chrysidid wasp that attacks a solitary host. Chemical camouflage, i.e. the acquisition of mimetic compounds from a solitary host by a parasitic species, might be problematic since, in contrast to parasites of social hosts, there is little opportunity to sequester cloaking chemicals. Social host species possess large nests and a large number of colony members that might serve as sources for the relevant compounds. Brood parasites of solitary brood caring Hymenoptera have rarely been studied in detail [53]. The only example of chemical camouflage in a brood parasite of a solitary species comes from *Nomada *bees. In some species of this genus, females have been reported to acquire mimetic odors by being perfumed by males during mating. Females of these species seemed not to elicit aggressive responses when encountered by host females of the genus *Andrena *[2]. Chemical cloaking in chrysidid wasps has not yet been reported.

With chemical camouflage being an unlikely option for a brood parasite of solitary species, chemical cloaking might evolve by synthesis of the compounds, i.e., chemical mimicry. For most chrysidids this is also unlikely since they attack a large number of different host species [22] with a varying composition of cuticular chemicals that is not compatible with an efficient chemical cloaking. However, *H. rutilans *is *de facto *monospecific in the study area and is, thus, predestined to evolve chemical mimicry. The behavioral observations show that cuckoo wasps do not regularly stay in nests for long periods. This makes a sequestration of host chemicals that are only available in relatively small amounts at the walls of the burrow rather unlikely. The assumption that the cloaking compounds are synthesized by *H. rutilans *females themselves is supported by details of the composition of chemicals on their cuticle. The occurrence of considerably proportion of the (*Z*)-7 isomers of the respective major components of the beewolf cuticle ((*Z*)-9-C25:1 or (*Z*)-9-C27:1) in the brood parasitoid but the lack thereof (at least in comparable proportions) in beewolf females contradicts an acquisition of the chemicals from their host. Thus, most probably cuckoo wasps produce at least some of the compounds on their cuticle by themselves.

In addition to the qualitative imitation of the hydrocarbons of its host, the cuckoo wasps might also employ a quantitative strategy to evade detection by beewolf females. Preliminary data suggest that *H. rutilans *(as well as several other chrysidids studied by us) have an overall low level of cuticular substances (Kroiss, Schmitt, Strohm, unpubl. data). Such a reduction in the amount of cuticular hydrocarbons as a means to avoid detection by the host has been suggested by Jeral et al. [54] in thievery ants (see also [41]). In fact, the background against which the cuckoo wasp is perceived (or rather not perceived) by the beewolf female is the wall of the nest burrow. This is contaminated with compounds from the cuticle of the nest owner due to the contact with its mandibles during excavation and with the tarsi and abdomen during processing of the excavated sand and movement inside the nest. Preliminary analyses suggest that the typical beewolf cuticular hydrocarbons can be sampled from the nest walls (Kroiss and Strohm, unpubl. data). Thus, the chemical traces or the presence of a cuckoo wasp whose hydrocarbon profile is sufficiently similar to the background might not be recognized by a beewolf female.

## Conclusion

*H. rutilans *might employ a combination of strategies to evade detection. If encountered in the nest, they run away or remain motionless. They possibly leave only very small amounts of decisive and traitorous substances in the nest. Most notably, the composition of their cuticular hydrocarbons is very similar to that of their host. Thus, *H. rutilans *females seem to be able to avoid detection when directly encountered by a beewolf female in the nest. Much more important, however, is the reduction of the conspicuousness of scent marks left in the nest burrow or on the bee during oviposition. This is to our knowledge the first reported evidence for chemical mimicry (sensu [19]) in a parasitoid of a solitary wasp.

## Methods

### Behavioral observations

#### Behavior outside the nest

We observed interactions between cuckoo wasps (*H. rutilans*)and beewolf females in the field in a beewolf nest aggregation on the Campus of the University of Würzburg. Over several years there were about 100 – 500 beewolf nests (easily detectable due to the characteristic nest mounds) and 50 – 500 *H. rutilans *females (determined by capture-mark-recapture methods [55], E. Strohm, unpubl. data). Behavioral interactions between beewolf females and cuckoo wasps at 24 focal nests (located on an area of about 10 × 5 m) were recorded for a total of 54 hours. We observed whether beewolf females showed any signs of disturbance or agonistic behavior when a cuckoo wasp was present in the vicinity of their nests. We recorded the following behaviors of cuckoo wasps and beewolf females: 1. Landing of *H. rutilans *on nest mound. 2. Time it stayed on nest mound (for stays > 4 sec.). 3. Whether the nest was open or closed. 4. Whether the cuckoo wasp entered the nest. 5. The time the cuckoo wasp stayed in the nest and whether the nest owner was at home or not. 6. Behavior of the cuckoo wasp during its stay outside the nest (running, sitting hiding, no exact durations were recorded). 7. Whether the cuckoo wasp tried to oviposit on a bee when a female returned with prey. 8. Whether and how a female responded to the presence of a cuckoo wasp when returning with prey.

#### Behavior inside the nest

##### a) Experiment I

The interaction between host and parasite inside the nest was investigated using observation cages in the laboratory (for details see [20]). These cages allow observation of the behavior of host and parasite in the main burrow. Beewolf females (N = 6), either from the laboratory population or from the field were kept individually in such cages and one cuckoo wasp that was caught in the field was introduced per cage (overall 7 *H. rutilans *females were used). Honey was provided *ad libitum *for both species. Honeybees were also provided *ad libitum *as prey for the beewolves. Since a pilot study revealed that the cuckoo wasps need a humid retreat, petri-dishes with a layer of moist sand and gravel were placed into the flight compartment of each cage and moistened daily. Observations of interactions in the nest burrow were carried out under dimmed red light that did not elicit any disturbance in either species. If a cuckoo wasp and a female were in a nest at the same time, we recorded the behavior of both.

##### b) Experiment II

To assess the significance of the cuticular hydrocarbons for the detection of *H. rutilans *beewolf female inside the nest, we established a recognition bioassay. We recorded the females' response towards paper discs treated with extracts. To make sure that beewolf females responded to the paper discs at all, we needed a positive control, i.e., extracts from a species that was recognized as an intruder and elicited strong responses by beewolf females. We used extracts of another chrysidid, *Chrysis viridula *(although it might have been preferable to use extracts of *H. nobile*, fresh specimens of this species were not available due to their rarity). *C. viridula *is a parasitoid of eumenid wasps and it may occur in the same habitat as *H. rutilans*. Its cuticular hydrocarbons differ considerably from the composition of the cuticular hydrocarbons of *H. rutilans *(Kroiss and Strohm, unpubl. data). Both cuckoo wasp species are very similar in size and the total amount of their cuticular hydrocarbons is alike (J Kroiss, E. Strohm, unpublished data). Cuticular hydrocarbons of females of *H. rutilans *and *Chrysis viridula *were extracted for 10 min in 0.5 ml distilled n-hexane (Fluka). The hexane of the cuticular extracts was evaporated under a stream of nitrogen at ambient temperature and the extract was redissolved in 100 μl hexane. An aliquot of 10 μl of an extract was applied onto a circular filter paper disc (diameter 6 mm. *H. rutilans*: N = 7, *C. viridula*: N = 8) and the solvent was allowed to evaporate for 5 min. As a negative control 10 μl of pure hexane were applied onto a filter paper disc and evaporated (N = 7). The filter paper discs were inserted into the main burrow of individual beewolf nests (N = 9) in observation cages (see above) one after the other in a randomized sequence. After introduction of the filter disc we continuously recorded the behavior of individual beewolf females using a voice recorder until the paper disc was evicted from the nest or until it was incorporated into excavated material in the nest. Beewolf females biting a paper disc or alert freezing directly at the paper disc when approaching it at any time during a trial were considered as evidence that a female has recognized the paper disc as something that differed from the background odor. The freezing behavior was distinctive and could not be observed as a spontaneous behavior. If one or several of these behaviors occurred the respective trials was considered as showing a response by the beewolf female. In contrast, trials during which females were walking over the paper disc without any response were classified as "no recognition". The prediction that extracts of *H. rutilans *elicited weaker responses than the positive control and similar responses as the negative control was tested by comparing the number of trials with and without recognition. The small sample size necessitated using Fisher's exact tests.

### Chemical analyses

We caught females of *H. rutilans *(N = 13) in the vicinity of beewolf nests on the campus of the University of Würzburg. Beewolf females (N = 7) and males (N = 8) were taken from the same field site in Würzburg or from a laboratory population that was bred from the same population. Honeybee foragers (*Apis mellifera carnica*) (N = 8) were caught from hives in the vicinity of the field site when leaving the nest. Females of *Hedychrum nobile *(Hymenoptera, Chrysididae) (N = 4) and its host, *Cerceris arenaria *(Hymenoptera, Crabronidae, subfamily Philantinae) (N = 4) were caught at a nesting aggregation near Vizzola Ticino, Italy. These two species are another host-parasitoid pair and served as an outgroup for *H. rutilans *and *P. triangulum *to control for phylogenetic relationships. All individuals were killed by freezing (1 h, -20°C). Chemicals on their cuticles were extracted for 10 min in 0.5 ml distilled n-Hexane (Fluka).

Capillary gas chromatography-mass spectrometry (GC-MS)-analysis was performed with a Fisons Instruments (Fisons, Engelsbach, Germany) GC 8000 Series coupled to a Fisons Instruments MD800 quadrupol mass detector. We used a DB-5MS fused silica capillary column (30 m × 0.25 mm i.d.; df = 0.25 μm) (J & W, Folsom, CA, USA). The GC was programmed from 60°C for 1 min then to 310°C for 10 min with a temperature increase of 5°/min, with 2 ml/min flow rate of helium gas. We chose a splitless injection mode (1 μl) at an injector temperature of 250°C and a splitless period of 60 sec. The mass spectrometer was operated in EI mode at 70 eV. The software Xcalibur for Windows was used for data acquisition.

Capillary Gas Chromatography – Fourier Transform Infrared Analysis (HRGC-FTIR). HRGC-FTIR spectra were obtained using an HP 5890 GC (Agilent Technologies, Böblingen, Germany) coupled to an FTS 575C Tracersystem (BioRad, Hercules, CA, USA). GC separation was performed using a DB-1 capillary column (30 m × 0.25 mm ID; df = 0.25 μm; J & W Scientific, Folsom, CA, USA). Temperature was programmed from 80 to 270°C with 4°C/min heating rate. Helium was used as carrier gas with a constant flow of 1–2 ml/min. Injection was carried out using a split/splitless injector at 250°C in the splitless mode for 60 sec. Injection volume was 0.1 μl. IR spectra were recorded by scanning 256 times in a frequency range from 4000 to 700 cm^-1 ^with a resolution of 1 cm^-1^. Data system was a Dell Optiplex GX110-PC with BioRad WinIR Pro (Version 2.7) Tracer Software and Sadtler IRSearchMaster.

The chemical structure of the components of the cuticular hydrocarbons was determined by comparing retention times and diagnostic ions of the mass spectra with purchased chemicals and the use of a commercial MS database (NIST 4.0). Methylalkanes were characterized using diagnostic ions and by determining Kovats indices according to the method of Carlson et al. (1998). The position of double bonds was determined by DMDS derivatisation [56]. The configuration of double bonds was revealed by HRGC-FTIR [57,58]. Some components could not be identified and for some alkenes the position of the double bond and its configuration could not be determined due to the small amounts on the cuticles. However, neither of the unidentified components occurs on beewolf females and cuckoo wasps. Thus, they do not confound the similarity between these two groups that are most important to our question. The alkenes listed in one line in Table 1 as the same compound for beewolves, cuckoo wasps and honeybees are most probably identical since their mass spectra and the retention times are identical. Thus, the comparison between beewolf females and cuckoo wasps is not confounded by the incompletely identified alkenes.

### Data analysis

The results of the behavioral observations are given as the mean ± SD and/or the median. For the recognition bioassay, we compared the number of individuals showing a response facing the paper disc between the treatments. Different treatments were compared using Fisher's exact test (two-tailed) using the program BIAS for Windows version 8.2 (epsilon-Verlag GbR, H. Ackermann, Frankfurt/Main, Germany). Since some of the females died before their response to all stimuli could be tested, sample sizes differ between the three stimuli.

Patterns of chemicals on the cuticle were analyzed by multivariate methods. Since we were interested in the similarity between *H. rutilans *females beewolf females, beewolf males, honeybee workers, *H. rutilans *females, and *H. nobile *and *C. arenaria *females, we performed a hierarchical cluster analysis to assess the pattern of similarity without *a priori *grouping. Furthermore, we conducted a discriminant analysis to test whether the groups are separated by discriminant functions. Due to the large number of peaks relative to the sample size the discriminant analysis might lead to confounded results with regard to the hypothesis tested. Thus, we reduced the number of variables for the discriminant analysis using principal component analysis (varimax rotation, eigenvalues > 1; 8 variables were extracted that represented 89% of the variance of the total sample).

Since relative peak areas of a sample are not statistically independent we transformed the data according to Aitchison ([59] see e.g. [60]). However, the original transformation procedure makes it necessary to exclude compounds that do not occur in all samples. Thus, peaks that are zero in some samples but are present in other samples would not have been considered. When analyzing whether groups can be separated by their profiles such a procedure is conservative. However, for the aim of this study, the exclusion of peaks that are not present in all samples would have erroneously increased the similarity between the groups and, thus, confounded the result. Therefore, we modified the transformation to avoid undefined values for peaks with an area of zero (log10((relative peak area/geometric mean relative peak area)+1)). The resulting variables were normally distributed. We used the squared Euclidean distance as a measure of distance for cluster analysis and between groups average linkage as the method for combining clusters. Analyses were calculated using SPSS 13.0.1.

## Competing interests

The author(s) declare that they have no competing interests.

## Authors' contributions

The manuscript was written by ES and JK. ES conceived the study, collected *H. rutilans *and *P. triangulum*, designed and conducted the statistical analysis. JK collected *H. nobile *and *C. arenaria*, performed the recognition bioassay, participated in the statistical analysis and the identification of the cuticular hydrocarbons of *H. nobile *and *C. arenaria*. GH participated in the quantitative analysis of the cuticular hydrocarbons. CLK conducted preliminary studies on the behavioral interaction. WB participated in the identification of the cuticular hydrocarbons in this preliminary study. PS participated in the identification of the cuticular hydrocarbons. TS identified the cuticular hydrocarbons. All authors read and approved the final manuscript.
